# Genetic Polymorphisms Affecting Ranibizumab Response in High Myopia Patients

**DOI:** 10.3390/pharmaceutics13111973

**Published:** 2021-11-20

**Authors:** David Blánquez-Martínez, Xando Díaz-Villamarín, Alba Antúnez-Rodríguez, Ana Pozo-Agundo, José Ignacio Muñoz-Ávila, Luis Javier Martínez-González, Cristina Lucía Dávila-Fajardo

**Affiliations:** 1 Pharmacy Department, Hospital Universitario de Ceuta, 51003 Ceuta, Spain; david.blanquez.sspa@juntadeandalucia.es; 2 Pharmacy Department, Hospital Universitario Clínico San Cecilio, 18016 Granada, Spain; 3 Instituto de Investigación Biosanitaria de Granada (ibs.Granada), 18016 Granada, Spain; alba.antunez@genyo.es (A.A.-R.); ana.pozo@genyo.es (A.P.-A.); 4 GENYO. Centre for Genomics and Oncological Research: Pfizer, University of Granada, Andalusian Regional Government, Genomics Unit, PTS Granada-Avenida de la Ilustración, 114-18016 Granada, Spain; luisjavier.martinez@genyo.es; 5 Ophthalmology Department, Instituto de Investigación Biosanitaria de Granada (ibs.Granada), Hospital Universitario Clínico San Cecilio, 18016 Granada, Spain; josei.munoz.sspa@juntadeandalucia.es; 6 Pharmacy Department, Instituto de Investigación Biosanitaria de Granada (ibs.Granada), Hospital Universitario Virgen de las Nieves, 18016 Granada, Spain; cristinal.davila.sspa@juntadeandalucia.es

**Keywords:** myopia, pharmacogenetic, genetic polymorphism, personalized medicine, ranibizumab, anti-VEGF, VEGFA, CFH, ARMS2

## Abstract

High myopia is an ophthalmic pathology that affects half of the young adults in the United States and Europe and it is predicted that a third of the world’s population could be nearsighted at the end of this decade. It is characterized by at least 6 diopters or axial length > 26 mm and, choroidal neovascularization (CNV) in 5 to 11% of cases. Ranibizumab is a recombinant humanized monoclonal antibody fragment. It is an anti-vascular endothelial growth factor (anti-VEGF) drug used in the treatment of CNV. Many genetic polymorphisms have been associated with interindividual differences in the response to ranibizumab, but these associations were not yet assessed among patients with high myopia and CNV. We performed a retrospective study assessing the association of genetic polymorphisms with response to ranibizumab in patients with CNV secondary to high myopia (mCNV). We included genetic polymorphisms previously associated with the response to drugs used in CNV patients (bevacizumab, ranibizumab, aflibercept, and photodynamic therapy (PDT)). We also included genetic variants in the *VEGFA* gene. Based on our results, *ARMS2* (rs10490924) and *CFH* (rs1061170) are associated with response to ranibizumab in high myopia patients; and, included *VEGFA* genetic polymorphisms are not associated with ranibizumab response in our population but might be related to a higher risk of CNV.

## 1. Introduction

Myopia is a pathology in which the image is cast in front of the retina instead of on it. It happens because the eyeball is too elongated, or in a minority of cases because of the increasing refractive index of the crystalline in nuclear cataract or cornea in keratoconus [1].

Myopia noted a rise worldwide over time. It affects about half of young adults in the United States and Europe, and it is even more prevalent in Asia. It has twice the prevalence compared to 50 years ago, and it is forecast that a third of the world’s population will be nearsighted at the end of this decade [2].

Myopia can be simple or high/pathological. A degree of myopic refractive error > −6 diopters or axial length (AL) of the eyeball < 26 mm characterizes the simple form, while a degree of myopic refractive error ≤ −6 diopters or AL ≥ 26 mm defines the high/pathological [1,3,4]. Severe myopia may be associated with increased risks of chorioretinal atrophy (23%), Fuch’s spot (5.2%), lacquer cracks (4.3%), retinal detachment (13%), cataracts (17%), glaucoma (13%) and mCNV (5% to 11%) [5,6].

This mCNV characteristic of high myopia is one of the complications that most compromises vision [7,8]. It is estimated to develop in 5% to 11% of these eyes with a higher prevalence in Asians than Caucasian populations [8,9]. It is the most common cause of CNV in people aged 50 years and younger [10,11] and the second most common cause of CNV after age-related neovascular macular degeneration (AMD) [8].

The appearance of retinal hemorrhages with or without exudation characterizes mCNV. In young individuals, it tends to appear as a small lesion close to the fovea while, in elderly patients, it tends to be more extensive and exudative [8,12,13,14]. Sometimes it can be resolved spontaneously without treatment, resulting in minimal consequences on eyesight [15]. But the vast majority of individuals will have a poor visual outcome, with visual acuity (VA) < 6/60 on the Snellen scale at 5-years from the onset of CNV in 89% of eyes and 96% at 10-years [10,16].

The treatment of mCNV began with laser-photocoagulation (LP), with good immediate results but long-term recurrence and growth of chorioretinal atrophy [17]. Surgery was also used, specifically removing the mCNV, but results were not good either, showing high rates of recurrence (>30%) and non-VA improvement (>35%) in high myopia patients [18]. In 2001, the results of the VIP study were published, based on the use of PDT with Verteporfin in mCNV, which showed no visual acuity worsening at 12 months follow-up [19] although, without significant differences in the primary outcome at 2-years [20]. In addition, the development of long-term chorioretinal atrophy was noted in another study [21].

The introduction of biological medicinal products against VEGF in the treatment of mCNV was a real breakthrough. Ranibizumab (Lucentis^®^) and aflibercept (Eylea^®^) are the only ones indicated in the treatment of mCNV among the currently available anti-VEGF drugs. Of those, ranibizumab was the first to be used among these patients, approved in 2013 by the European Medicines Agency (EMA) and 2017 by the Food and Drug Administration (FDA), based on the results of the RADIANCE study [22]. This one and other phase-III randomized controlled clinical trials [23,24] have shown that ranibizumab leads to good efficacy and safety results in patients with mCNV, with a significant and sustained gain of VA. Hamilton R.D. et al. showed a mean (±SD) VA letter changes of +9.7 (±17.99) from 49.5 (±20.51) among treatment-naïve patients and +1.5 (±13.15) from 58.5 (±19.79) among prior ranibizumab-treated patients [24]. Furthermore, to treat most of the patients with mCNV, it was required a relatively low number of injections, with a mean (SD) of 3.0 (±1.58) injections among treatment-naïve patients resulting in mean (SD) VA changes of +15.0 (±14.70) and +7.7 (±19.91) when 1–2 or 3–4 injections are used respectively [24].

**Figure 1:**
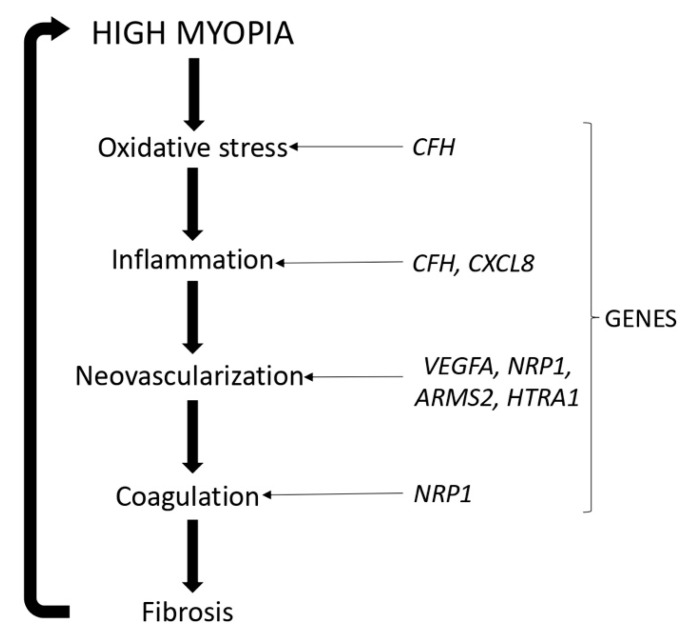
High myopia and related genes.

To assess the difference in the efficacy of ranibizumab between Asian and Caucasian patients, a subgroup analysis of the RADIANCE study was performed [25]. Asians showed higher best-corrected visual acuity (BCVA) than Caucasians, with a lower median number of injections. It might suggest the existence of genetic variants associated with the response of patients to ranibizumab treatment (Figure 1).

Many genetic polymorphisms are associated with variable responses to drugs used to treat CNV (anti-VEGF drugs and PDT). In AMD patients, the *CXCL8-251* (rs4073) AA genotype, and minor allele (A), were associated with non-response to bevacizumab treatment in the European population [26]. The *CFH Y402H* (rs1061170) single nucleotide polymorphism (SNP) was related to higher doses and decreased response to bevacizumab [27,28]. To greater improvement in mean VA when treated with ranibizumab [29], and decreased response to PDT [30]. The *NRP1* (rs2070296) T allele [27] and *ARMS2* (rs10490924) TT genotype were related to decreased response to bevacizumab and ranibizumab, respectively [31]. The *HTRA1-625* A/G (rs11200638) genetic variant was associated with decreased response to bevacizumab [31]. Also, many genetic polymorphisms in the *VEGFA* gene were associated with interindividual differences in the responses to anti-VEGFA drugs or PDT among AMD patients [27,29,32]. Among CNV and/or polypoidal choroidal vasculopathy patients, usually considered a subtype of CNV, also there are SNPs associated with variable response to ranibizumab, bevacizumab or PDT as *HTRA1*-625 A/G (rs11200638), *F13A1* (rs5985) *CFH I62V* (rs800292), *CFH Y402H* (rs1061170) and, especially, *ARMS2 A69S* (rs10490924) [33].

As we can see, no studies have assessed the association of genetic variants with interindividual differences in the responses to ranibizumab among high myopia patients despite ranibizumab being commonly used to treat this pathology.

This study aims to evaluate the association of *VEGFA* variants with interindividual differences in the responses to ranibizumab. It also aims to assess the association of genetic polymorphisms previously associated with the response to treatments used in patients with CNV (anti-VEGF drugs and PDT), excluding drug-SNP interactions in non-ophthalmic indications (cancer, hypertension). Furthermore, as a secondary objective, it is intended to characterize these genetic polymorphisms in our population and evaluate their possible association as genetic markers of CNV.

## 2. Materials and Methods

A retrospective study including patients with high myopia and CNV treated with ranibizumab between 2014 and 2019 in San Cecilio University Hospital (Granada, Spain). We studied the association of genetic polymorphisms with differences in response to treatment from baseline (BL) up to 1 and 6 months of follow-up.

The Research Ethics Committee of Granada approved the study (0085-N14; 26 May 2014). We obtained written informed consent from all participants and we followed the principles of the Declaration of Helsinki.

### 2.1. Inclusion/Exclusion Criteria

The inclusion criteria were: 1. Diagnosed high myopia and CNV, 2. Treatment with ranibizumab by prescription from ophthalmologists at our hospital, 3. Treated at our hospital, 4. At least six months follow-up with available medical records.

The exclusion criteria were: 1. History of intraocular surgery except for cataract surgery, 2. cataract surgery during the follow-up period, 3. Previous anti-VEGF treatment and 4. Presence of other ocular pathology that may influence the BCVA.

We considered high myopia: AL ≥ 26.00 mm or a spherical equivalent refractive error of ≥−6.0 diopters in phakic eyes and documentation of CNV by optical coherence tomography (OCT) or OCT angiography. If patients underwent bilateral anti-VEGF treatment, both eyes were chosen for analysis.

Furthermore, we recruited a control group with high or pathological myopia but with neither CNV nor other associated pathology and not treated with ranibizumab to control the possible association of genetic polymorphisms with the disease and not with the drug response. The inclusion criteria in this group were: 1. Diagnosed high myopia, 2. Age > 18. Exclusion criteria: 1. Another ophthalmic pathology except for cataract surgery, including diagnosis of CNV.

### 2.2. Patients Management

All patients received a 0.5 mg intravitreal dose of ranibizumab. All injections were performed under sterile conditions, and prophylactic topical antibiotics were applied from a few days before to a week after injection. Anti-VEGF therapy was started by a single injection. At baseline and after the first injection, ophthalmologic examinations, including measurement of BCVA, indirect ophthalmoscopy, slit-lamp biomicroscopy with a contact lens by a retina specialist, and OCT were performed at scheduled visits with 1-month intervals. The reinjection criteria included any of the following findings: presence or recurrence of fluid indicative of active CNV on OCT images, new subretinal hemorrhage, and worsening of subjective symptoms, such as metamorphopsia, central scotoma, paracentral scotoma, or VA loss. To recruit both the treatment and control groups, a nurse daily reviewed all the medical records of the patients cited in the ophthalmology unit because of the follow-up of their pathology, and we selected all patients meeting the inclusion/exclusion criteria. At this moment, the nurse asked the patient to sign the informed consent and took four saliva samples with sterile cotton swabs.

### 2.3. Data Management

It was collected the following data from the clinical records of patients: medical record number, age, gender, date of diagnosis, date of initiation of treatment, affected eye, LA, refractive error, location of CNV, baseline VA and VA at 1 and 6 months.

As the primary endpoint of the study, BCVA was measured with the Snellen optotype, in meters, and it was converted to a logarithm of the minimum angle of resolution (logMAR). The increase in logMAR was measured from BL and after 1 and 6 months. The evolution of the patients was ranked, according to the change in logMAR, in IMPROVEMENT (YES/NO) or WORSENING (YES/NO). IMPROVEMENT was a decrease, of at least 0.1 in the logMAR scale, from BL during the follow-up time (1 or 6 months). On the contrary, WORSENING was an increase, of at least 0.1 in the logMAR scale, during this time.

### 2.4. Procedures for the Inclusion of Genetic Variants in the Study

To include genetic variants that could affect the response that patients have to ranibizumab, we considered including genetic variants that had been previously associated with the response to treatments used in patients with high myopia and CNV (bevacizumab, ranibizumab, aflibercept, and PDT), if this association had been found in patients with any ophthalmic pathology.

To do this, we searched in PharmGKB the following terms: ranibizumab, bevacizumab, aflibercept, and PDT. We then considered for inclusion all the drug-SNP interactions reported as clinical annotations with ophthalmic-related phenotypes, thus excluding those drug-SNP interactions related to phenotypes such as “breast neoplasm” or “hypertension”, among others. Since ranibizumab is an anti-VEGF drug, we also considered including any genetic variant in the *VEGFA* gene previously associated with response to any other drug. To do this, we also searched in PharmGKB the term “VEGFA” (gene), and we considered for inclusion all resulting drug-gene interactions reported as clinical annotations.

Table 1 shows all the genetic variants found after this search, thus considered for inclusion in our study. Among these variants, we studied possible linkage disequilibrium. Among those variants that presented some linkage, we chose the one with higher frequency in our population. Finally, we excluded variants with Minor Allele Frequency (MAF) lower than 10 or linked to other positions.

### 2.5. DNA Extraction and Genotyping

For genotyping, DNA was isolated from saliva using standard procedures. DNA extraction was carried out according to the method by Freeman et al. [34], a non-organic (proteinase K and salting-out) protocol with modifications described by Gomez-Martín A. et al. [35]. The SNPs were genotyped using KASP assay technology (LGC Genomics, Hoddesdon, Hertfordshire, UK) and analyzed with KlusterCaller Software from LGC Genomics.

### 2.6. Statistical Analysis

First, we performed a descriptive analysis of clinical parameters recorded from patients (Table 2). Then we calculated the distribution (number of patients and percentage) of genotypes and MAF of each genetic variant for the total number of patients, control, and study groups (Table 3). Here we also studied the Hardy-Weinberg (H-W) equilibrium of each genetic polymorphism for each group and compared the distribution of genotypes between the control and study groups. This comparison allowed us to assess the association of each SNP with CNV.

As main results, we studied the association between each genetic polymorphism and BCVA (logMAR) improvement or worsening at 1- and 6-months follow-up. We performed an allele comparison analysis and a genotype analysis using each genetic model (recessive, dominant, co-dominant, over-dominant, and log-additive).

For the comparison of the distribution of genotypes between control and study group, and main results, we used the Chi-square. We calculated the odds ratio (OR) and *p*-values. *p*-values < 0.05 were considered statistically significant. We also calculated the Akaike information criterion (AIC) and Bayesian information criterion (BIC) for each genetic model and SNP-response association study. We chose for the main results genetic models showing lower AIC and BIC.

The descriptive analysis of clinical parameters, MAFs, genotypic distribution, and its comparison among groups was performed using R commander. The H-W equilibrium analysis, linkage disequilibrium in *VEGFA*, association study of VEGFA hapotypes with response and main results were conducted using the SNPstats online tool [36].

The sample size calculation was based on previous research articles evaluating the influence of considered genetic polymorphisms on ranibizumab response on patients with other pathologies [27,28,29,30,31,32,33]. Furthermore, we recruited the total number of patients with high myopia and treated them with ranibizumab in our hospital for five years. Both sample size and statistical influence of SNPs on genetic diseases were studied in several works which investigated the impact on pathological phenotypes [37,38,39,40].

## 3. Results

In total, between 2014 and 2019 in our hospital, n = 100 patients and n = 113 eyes were diagnosed with CNV secondary to high myopia and treated with ranibizumab, thus eligible for this study. DNA concentration from one patient (n = 1 eye) was not enough to do the needed genotypes. Finally, n = 99 patients and n = 112 eyes were recruited and included in the study, with a mean age of 57.5 ± 13.9 years and 75% women. Two out of three eyes presented juxta-foveal development of CNV, n = 30 (26.8%) sub-foveal and n = 8 (7.1%) extra-foveal location. Almost all eyes (92%) were not previously treated with LP or PDT because of their high myopia, while n = 8 (7.1%) and n = 1 (0.9%) had been treated with LP and PDT respectively. Mean spherical equivalent refractive error was 12.1 ± 5.4, mean BCVA (logMAR) = 0.62 ± 0.48 at baseline; 0.42 ± 0.41 at 1 month and 0.36 ± 0.36 at 6 months follow-up (Table 2).

In the control group, we recruited n = 116 patients and n = 219 eyes, with mean age 57.5 ± 15.1 years and 68% women. Furthermore, n = 7 eyes could not be assessed because of the following causes: DNA concentration from 4 patients (n = 4 eyes) was not enough to do the needed genotypes. Saliva samples from 2 patients (n = 2 eyes) were lost, and one patient (n = 1 eye) was diagnosed with mCNV one month after being recruited, thus crossed from the control to the treatment group.

Among treated eyes, 67% (n = 75) improved and 7.1% (n = 8) worsened at 1-month follow-up, and 71.4% (n = 80) improved and 12.5% (n = 14) worsened at 6-months.

### 3.1. Genotypic Distribution, H-W Equilibrium, and Association of Genetic Variants with CNV

All the genetic variants included in the analysis showed MAFs higher than 0.1. Studied SNPs in *ARMS2*, *CFH,* and *F13A1* showed significant differences neither in the H-W equilibrium analysis nor the comparison of the genotypic distribution between control and study groups (Table 3).

Considering the total patients genotyped (control + study; N = 215), only variants in *VEGFA* showed a deviation from the H-W equilibrium. If we look at the genotypic distribution of this gene in the control or study groups (Table 3), we see that deviations from the H-W equilibrium happen in the study group (rs699947, rs3025000 and rs2010963) and no significant differences were found in the control group. Furthermore, one of these genetic polymorphisms (rs3025000) showed a *p*-value = 0.063 in the comparison of genotypic distribution between study and control group, and most of the studied SNPs in this gene (rs25648, rs699947, rs3025000, rs1570360 and rs2010963) were found to be almost full linked among them (*p*-value < 1 × 10^−5^; D’ > 0.95; r > 0.25) (Figure S1).

Regarding *CXCL8* (rs4073), this SNP showed a deviation from H-W equilibrium (*p* = 0.01) in the control group with n = 32 (27.6%) individuals carrying A/A genotype (wildtype), n = 43 (37.1%) A/T and n = 41 (35.3%) T/T. We also found a trend for association (*p* = 0.075) in the comparison between genotypic distribution between control and study group.

Finally, regarding *NRP1* (rs2070296), this SNP also showed statistically significant differences in the distribution of genotypes among control and study groups (*p* = 0.011), but without showing deviations from H-W equilibrium.

As we can see, *F13A1* (rs5985), *ARMS2* (rs10490924), *CFH* (rs1061170) and *VEGFA* (rs3025040) are the only SNPs not showing significant (*p* > 0.05) or close to significance (*p* < 0.1) differences about their genotype distribution among control and study groups, or deviations from the H-W.

### 3.2. Association of Genetic Polymorphisms with Response

#### 3.2.1. Allele Association Study with Response

In the allele association study with response, the *CFH* (rs1061170) was the only SNP showing significant results at 1-month follow-up (Table 4A). The C allele was found to protect against BCVA worsening (OR = 0.13; 95%CI = 0–0.91; *p* = 0.025) compared to T allele.

At 6-months follow-up (Table 4B), we found that *ARMS2* (rs10490924) G allele is associated with increased BCVA improvement (OR = 1.95; 95%CI = 0.98–3.83; *p* = 0.037) and showed a trend for the association with worsening (*p* = 0.073) compared to the T allele.

Regarding the *CFH* (rs1061170) in the same follow-up period (6-months), the C allele showed close to significant results for its association with increased BCVA improvement (OR = 1.82; 95%CI = 0.9–3.8; *p* = 0.075) compared to T allele.

Table 4 shows results about the allele association study with response to ranibizumab (BCVA improvement or worsening) in high myopia patients of included genetic polymorphisms. Table 4A shows results at 1-month follow-up and Table 4B at 6-months follow-up.

#### 3.2.2. Genotype Association Study with Response

In the genotype association study with the response at 1-month follow-up (Table 5A), the *CFH* (rs1061170) was again the only, among studied SNPs, associated with response. The TT genotype showed higher rates of worsening (recessive model: TT vs. CT-CC; OR = 8.83; 95%CI = 1.05–74.3; *p* = 0.013).

At 6-months follow-up (Table 5B), the genotype’s association study with response showed that *ARMS2* (rs10490924) GG genotype is associated with increased BCVA improvement (OR = 2.44; 95%CI = 1.05–5.63; *p* = 0.035) and decreased BCVA worsening (OR = 0.26; 95%CI = 0.08–0.90; *p* = 0.024) compared to GT or TT genotypes. It also showed that *CFH* (rs1061170) CC genotype is associated with increased BCVA improvement (*p* = 0.005) compared to TC or TT genotypes, but we did not find patients carrying CC genotype and non-BCVA improvement.

Table 5A,B indicate at 1- and 6-months follow-up respectively, the results of the genotype association study with BCVA improvement/worsening of *CFH* (rs1061170) and *ARMS2* (rs10490924). Supplementary Tables S1 and S2 show these results for the other included SNPs.

Regarding the association study of *VEGFA* variants with response, we found no significant results (Supplementary Table S3).

Based on AIC and BIC, the dominant and recessive models are respectively the best explaining the association of *ARMS2* (rs10490924) and *CFH* (rs1061170) SNPs with the response to ranibizumab in patients with mCNV.

Summarizing, among our patients and studied SNPs, the *CFH* (rs1061170) and *ARMS2* (rs10490924) were the only associated with the response at 1-month or 6-months follow-up in the allele or genotype association study. The *CFH* (rs1061170) C allele is associated with protection against BCVA worsening at 1-month and with BCVA improvement at 6-months; TT genotype is associated with worsening at 1-month, and CC genotype with improvement at 6-months. Regarding *ARMS2* (rs10490924), the G allele, and GG genotype, are associated with BCVA improvement at 6-months.

Among those other studied genetic polymorphisms, we found no genotypes (see Supplementary Tables S1 and S2) or alleles (Table 4A,B) associated with ranibizumab response in patients with high myopia regardless of the follow-up time.

## 4. Discussion

Ranibizumab and bevacizumab are both anti-VEGF drugs used in the treatment of mCNV among other pathologies. The influence of genetic polymorphisms on anti-VEGF drugs response has been widely studied in patients with AMD, especially for bevacizumab [41,42]. In this study, we assessed in patients with mCNV, if ten genetic variants in the *VEGFA* gene and those genetic polymorphisms previously related to interindividual differences in the response to drugs used in the treatment of CNV (anti-VEGFA drugs and PDT), are associated with ranibizumab efficacy.

We still had limitations in this study. It is a retrospective study; we recruited a control group, but we did not collect data about BCVA in this group since this was only to control the possible association of genetic polymorphisms with CNV, not with ranibizumab response. Also, the study cohort is low and it should be increased in further studies. We recruited only n = 100 patients in 5 years period of recruitment but these were the total patients treated with ranibizumab with high myopia in our hospital. Because of this, *CFH* (rs1061170) association with ranibizumab response at 6 months could not be confirmed since we did not find at least one patient carrying CC (recessive homozygous) genotype and non-BCVA improvement (Table 5B). In the same regard, this impeded to perform a multivariant analysis considering combined genetic and clinical parameters affecting ranibizumab response. Following in this regard, we included both eyes of patients with bilateral treatment considering that they might progress in a different way depending on the expression/silencing of PGx variants.

This is an observational study, and we did not conduct a clinical trial assessing the usefulness of tailoring anti-VEGF treatment depending on PGx. But this would not be ethical since not even these genetic polymorphisms had been related to interindividual differences on ranibizumab response.

Our results support the need for further studies improving these limitations, thus performing a clinical trial including combined PGx, clinical parameters (e.g., previous treatments as LP, PDT), in a multivariant analysis, and in a larger cohort including patients from different populations.

We considered for inclusion in this study only genetic polymorphisms previously associated with response to drugs used in AMD or CNV patients and we did not include in the analysis other SNPs found in those genes if they were not associated in this regard. On the other hand, we found only one SNP, in *CXCL8*, the rs1126647, previously related to drug response, but this had been associated with sunitinib response [43], not with any treatment used in AMD or CNV. No other SNP found in assessed genes were related to any other drug response when this study was performed.

Also, we calculated BCVA improvement/worsening based on BCVA (logMAR) change from BL to 1 or 6-months follow-up. Results about the association of SNPs with mean logMAR change (quantitative crude parameter) were not considered and this should be addressed in future studies. As we can see in Table 2, mean logMAR at baseline and 6 months follow-up were 0.63 ± 0.48 and 0.36 ± 0.39 respectively, thus interindividual variability about this parameter may lead to confusing results about the association between genetic polymorphisms and response.

### 4.1. ARMS2 (rs10490924) and CFH (rs1061170)

The *ARMS2* gene encodes a protein with unclear biological function [44] expressed in the choroid, monocytes, and microglia cells, mediating its activity against apoptotic cells by complement activation [45], so genetic polymorphisms in this gene may lead to decreased apoptotic cell clearance, influencing CNV development [46] and anti-VEGF drugs response as ranibizumab.

This SNP had been previously related to anti-VEGF drugs response with inconclusive results. Ku Kang H. et al. [47] found that patients with the GT and TT genotype had greater improvements in visual acuity at 6 months of treatment with bevacizumab in the east Asian population, as compared to those with the GG genotype, but reporting n = 8 (10.7%) of patients carrying GG genotype, which does not accord with MAFs for this SNP reported in large datasets [48,49]. Also, many studies found no association with response [27,50,51] but considering different follow-up periods, endpoints, and none of them in high myopia patients. On the other hand, a study by Tian J. et al. [31] reported an association between TT genotype with decreased response to bevacizumab in people with Macular Degeneration.

Among our patients, *ARMS2* (rs10490924) showed to be associated with ranibizumab response at 6-months follow-up. The GG (dominant homozygous) genotype was related to increased response (greater BCVA improvement and decreased worsening) compared to GT or TT genotypes, G allele was associated with greater BCVA improvement, and showed a *p*-value = 0.073 in the association study with decreased worsening compared to T allele (minor). We did not find differences about genotypic distribution among treated and control group or deviation from H-W equilibrium. On the other hand, we did not find significant differences at 1-month follow-up.

The *CFH* gene encodes the complement factor H expression, which inhibits components in the complement cascade related to inflammation and drusen appearance [52]. Changes in the *CFH* gene may result in a malfunctioning CFH, rendering it unable to inhibit the complement cascade [53], especially its affinity to C-reactive protein (CRP), enhancing CRP levels in the choroid. This may lead to increased levels of VEGF resulting in neovascularization [54], thus genetic polymorphisms in *CFH* may influence ranibizumab response, especially in CNV patients.

Previous studies found inconclusive results about the association of *CFH* (rs1061170) with response to anti-VEGF drugs in AMD patients [27,47,51], but no studies were done with high myopia patients.

Among our patients at 1-month follow-up, the *CFH* (rs1061170) C allele was found to protect against BCVA worsening (OR = 0.13; 95%CI = 0–0.91; *p* = 0.025) and TT genotype showed higher rates of worsening (recessive model: TT vs. CT-CC; OR = 8.83; 95%CI = 1.05–74.3; *p* = 0.013). At 6-months the C allele showed a trend for the association with increased BCVA improvement (*p* = 0.075) compared to T allele and CC genotype associated with increased BCVA improvement (*p* = 0.005) compared to TC or TT genotypes, but we did not find patients carrying CC genotype and non-BCVA improvement, so we cannot confirm this association.

As we can see, both *ARMS2* (rs10490924) and *CFH* (rs1061170) showed quite similar results at 1 or 6-months but differences. These discordances about the association with response depending on follow-up time might be explained by the lack of response at one month or by the size of the hemorrhage produced by the neovascularization. Some patients had more extensive bleeding at baseline than others. These patients may have a favorable genetic profile but could have a slower response to treatment with a slower recovery of BCVA.

### 4.2. VEGFA Genetic Polymorphisms

*VEGFA* gene is a member of the PDGF/VEGF growth factor family which encodes a heparin-binding protein. This growth factor induces proliferation and migration of vascular endothelial cells and it is essential for both physiological and pathological angiogenesis.

Genetic variants in *VEGFA* included in this study were not associated with ranibizumab response among our patients (see Supplementary Tables S1 and S2). Most of them (rs25648, rs699947, rs3025000, rs1570360 and rs2010963) were found to be almost full linked (*p*-value < 1 × 10^−5^; D’ > 0.95; r > 0.25) (see Supplementary Figure S1), among these five SNPs, three were not in H-W equilibrium (*p* < 0.05; rs699947, rs3025000 and rs2010963) and remaining two (rs25648 and rs1570360) were almost in disequilibrium (*p* < 0.1). H-W equilibrium deviations might be explained by linkage disequilibrium.

Furthermore, *VEGFA* (rs3025000) showed almost significant differences about their genotype distribution among control and study groups (*p* = 0.063). We found n = 70 (60.3%) individuals carrying the *VEGFA* (rs3025000) CC genotype, n = 8 (6.9%) with TT and MAF = 23.3% in the control group, compared to n = 52 (52.5%), n = 17 (17.2%) and MAF = 32.3% in the study group. This suggests its association with higher risk of CNV.

The only SNP in *VEGFA* included in this study in the H-W equilibrium, without differences between control and study groups and not linked with others was the *VEGFA* (rs3025040), but this was neither associated with response to ranibizumab (see Table 4A,B, and Supplementary Tables S1 and S2).

Anyway, *VEGFA* variants should be further studied for their associations with drugs responses, characterizing relevant variants, and considering interactions among genetic polymorphisms.

### 4.3. Other Genetic Polymorphisms Included in This Study

*CXCL8* encodes a protein member of the CXC chemokine family which acts as a major mediator of the inflammatory response. The encoded protein is commonly referred to as interleukin-8, secreted by mononuclear macrophages, neutrophils, eosinophils, T lymphocytes, epithelial cells, and fibroblasts. Among our patients, the *CXCL8* (rs4073) was not associated with ranibizumab response and it showed a deviation from the H-W equilibrium (*p* = 0.01) and was almost significant (*p* = 0.075) differences about genotype distribution between control and study groups.

The deviation from H-W equilibrium and genotypic distribution among groups might mean that this SNP is related, in some way, with the absence of CNV, but further studies are needed and other reasonings as the low number of patients carrying this variant might explain this regard.

On the other hand, *NRP1* (rs2070296) showed significant differences (*p* = 0.011) for the genotypic distribution among groups without deviations from H-W equilibrium which suggests its association with CNV development.

*F13A1* (rs5985) showed no significant association with response neither in the allele nor genotype analysis, no differences among groups or deviations from H-W equilibrium.

## 5. Conclusions

CNV is a complication that may occur in patients with AMD or high myopia. Patients presenting high myopia and CNV are usually treated with anti-VEGF drugs (ranibizumab, bevacizumab, and aflibercept) or PDT. Among AMD patients, some genetic polymorphisms showed differences in the response to these drugs, especially bevacizumab, resulting in different levels of evidence. In contrast, it is difficult to find studies evaluating the influence of these genetic polymorphisms on the response to ranibizumab regardless of the pathology, and no one reports results about patients with high myopia.

Based on our results, *ARMS2* (rs10490924) and *CFH* (rs1061170) SNPs are associated with response to ranibizumab in high myopia patients with CNV.

The *ARMS2* (rs10490924) G allele and GG genotype lead to a better response to this drug at 6-months follow-up. By contrast, the *CFH* (rs1061170) T allele and TT genotype are associated with higher rates of BCVA worsening at 1-month follow-up among these patients.

Finally, included *VEGFA* genetic polymorphisms are not associated with ranibizumab response in high myopia patients but these might be related to a higher risk of CNV.

## Figures and Tables

**Table 1:** Genetic polymorphisms considered for inclusion.

Gene	Major Nucleotide Variation	Rs	MAF	Response-Related Drug	Exclusion Criteria	Included
*CXCL8*	A > T	rs4073	42/58	Bevacizumab	-	Yes
*NRP1*	C > T	rs2070296	84/16	Ranibizumab	-	Yes
*ARMS2*	G > T	rs10490924	81/19	Bevacizumab	-	Yes
*CFH*	C > T	rs1061170	36/64	Bevacizumab/ Ranibizumab/PT	-	Yes
*HTRA1*	G > A	rs11200638	81/19	Bevacizumab/ Ranibizumab	Linked: rs10490924 (*ARMS2*)	No
*F13A1*	C > A	rs5985	76/24	PT	-	Yes
*VEGFA*	A > G	rs13207351	50/50	Other	Linked: rs699947	No
*VEGFA*	* (INDEL)	rs144854329	(INDEL) 50/50	Other	Linked: rs699947	No
*VEGFA*	A > G	rs1570360	32/68	Other	-	Yes
*VEGFA*	C > G	rs2010963	31/69	Bevacizumab/ Ranibizumab	-	Yes
*VEGFA*	C > T	rs25648	83/17	Other	-	Yes
*VEGFA*	C > T	rs3025000	71/29	Bevacizumab/ Ranibizumab	-	Yes
*VEGFA*	C > T	rs3025039	88/12	Other	Linked: rs3025040	No
*VEGFA*	C > T	rs3025040	88/12	Other	-	Yes
*VEGFA*	G (INDEL)	rs35864111	(INDEL) 50/50	Other	Linked: rs699947	No
*VEGFA*	C > T	rs6900017	91/9	Other	MAF: 91/9	No
*VEGFA*	A > C	rs699947	50/50	Ranibizumab	-	Yes
*VEGFA*	C > T	rs833061	50/50	Other	Linked: rs699947	No
*VEGFA*	T > C	rs833069	69/31	Ranibizumab	Linked: rs2010963	No
*VEGFA*	A > G	rs879825	91/9	Other	MAF: 91/9	No
*VEGFA*	T > C	rs9369421	91/9	Other	Linked: rs879825	No

Rs: Reference single nucleotide polymorphism; MAF: Minor allele frequency; PT: Photodynamic therapy. *: GGTCCCACTCTTCCCACAGG > GG.

**Table 2:** Baseline characteristics of patients.

Variable	Ranibizumab Mean ± SD or n (%)	
Study		Control
**Baseline Characteristics**		
Total eyes (n)	112	219
Mean age (years)	57.5 ± 13.9	57.5 ± 15.1
Sex (Male:Female,%)	25:75	32:68
Mean SERE (Diopters)	12.1 ± 5.4	12.3 ± 4.9
Mean AL (mm)	28.8 ± 2.1	28.3 ± 1.9
Affected eye		
RE	61 (54.5)	
LE	51 (45.5)	
CNV Location		
Subfoveal	30 (26.8)	
Juxtafoveal	74 (66.1)	
Extrafoveal	8 (7.1)	
Previous treatment		
None	103 (92)	
LP	8 (7.1)	
PDT	1 (0.9)	
BCVA (logMAR) at BL	0.62 ± 0.48	
**1-Month Follow-up Characteristics**		
BCVA (logMAR)	0.42 ± 0.41	
BCVA change (logMAR)	−0.21 ± 0.28	
BCVA improvement:		
Improvement	75 (67.0)	
Non improvement	30 (26.8)	
Worsening	8 (7.1)	
**6-Month Follow-up Characteristics**		
BCVA (logMAR)	0.36 ± 0.36	
BCVA change (logMAR)	−0.26 ± 0.35	
BCVA improvement:		
Improvement	80 (71.4)	
Non improvement	18 (16.1)	
Worsening	14 (12.5)	

SERE: Spherical equivalent refractive error; BCVA = best-corrected visual acuity; BL: Baseline; CNV = choroidal neovascularization; AL = axial length; LE = left eye logMAR = logarithm of the minimum angle of resolution; LP = laser photocoagulation; PDT = photodynamic therapy; RE = right eye; SD = standard deviation.

**Table 3:** Genotype distribution, minor allele frequency and Hardy-Weinberg equilibrium analysis for each studied SNP in our populationequilibrium, thus being candidates to influencing ranibizumab response; while CXCL8 (rs4073), NRP1 (rs2070296) and VEGFA (rs3025000, rs25648, rs699947, rs1570360, rs2010963) variants, have shown differences in this regard.

SNP	TOTAL N = 215					Control Group n = 116					Study Group n = 99					Control vs. Study
	Genotypes N (%)			MAF Allele: %	H-W	Genotypes n (%)			MAF Allele: %	H-W	Genotypes n (%)			MAF Allele: %	H-W	*p*-Value
	Wt	Het	Hom			Wt	Het	Hom			Wt	Het	Hom			
*CXCL8* A > T rs4073	52 (24.2)	95 (44.2)	68 (31.6)	A: 46.3	0.1	32 (27.6)	43 (37.1)	41 (35.3)	A: 46.1	0.01	20 (20.2)	52 52.5)	27 (27.3)	A: 46.5	0.69	0.075
*NRP1* C > T rs2070296	132 (61.4)	73 (34.0)	10 (4.6)	T: 21.6	1	61 (52.6)	47 (40.5)	8 (6.9)	T: 27.2	1	71 (71.7)	26 (26.3)	2 (2.0)	T: 15.2	1	0.011
*F13A1* C > A rs5985	133 (61.9)	71 (33.0)	11 (5.1)	A: 21.6	0.69	72 (62.1)	36 (31.0)	8 (6.9)	A: 22.4	0.28	61 (61.6)	35 (35.4)	3 (3.0)	A: 20.7	0.56	0.394
*ARMS2* G > T rs10490924	123 (57.2)	77 (35.8)	15 (7.0)	T: 24.8	0.58	67 (57.8)	42 (36.2)	7 (6.0)	T: 24.1	1	56 (56.6)	35 (35.4)	8 (8.1)	T: 25.8	0.44	0.842
*CFH* C > T rs1061170	23 (10.7)	83 (38.6)	109 (50.7)	C: 30.0	0.26	12 (10.3)	41 (35.3)	63 (54.3)	C: 28.0	0.17	11 (11.1)	42 (42.4)	46 (46.5)	C: 32.3	0.82	0.504
*VEGFA* C > T rs25648	156 (72.5)	50 (23.3)	9 (4.2)	T: 15.8	0.07	85 (73.3)	27 (23.3)	4 (3.4)	T: 15.1	0.29	71 (71.7)	23 (23.2)	5 (5.1)	T: 16.7	0.14	0.841
*VEGFA* A > C rs699947	60 (27.9)	94 (43.7)	61 (28.4)	A: 49.8	0.08	32 (27.6)	57 (49.1)	27 (23.3)	C: 47.8	0.85	28 (28.3)	37 (37.4)	34 (34.3)	A: 47.0	0.02	0.135
*VEGFA* C > T rs3025000	122 (56.8)	68 (31.6)	25 (11.6)	T: 27.4	<0.01	70 (60.3)	38 (32.8)	8 (6.9)	T: 23.3	0.43	52 (52.5)	30 (30.3)	17 (17.2)	T: 32.3	<0.01	0.063
*VEGFA* A > G rs1570360	29 (13.5)	87 (40.5)	99 (46.0)	A: 33.7	0.17	16 (13.8)	53 (45.7)	47 (40.5)	A: 36.6	0.84	13 (13.1)	34 (34.3)	52 (52.5)	A: 30.3	0.06	0.184
*VEGFA* C > T rs3025040	164 (76.3)	48 (22.3)	3 (1.4)	T: 12.6	1	87 (75.0)	27 (23.3)	2 (1.7)	T: 13.4	1	77 (77.8)	21 (21.2)	1 (1.0)	T: 11.6	1	0.839
*VEGFA* C > G rs2010963	26 (12.1)	76 (35.3)	113 (52.6)	C: 29.8	0.03	64 (55.2)	43 (37.1)	9 (7.8)	C: 26.3	0.63	49 (49.5)	33 (33.3)	17 (17.2)	C: 33.8	0.01	0.108

SNP: Single Nucleotide Polymorphism; Wt: Wildtype genotype; Het: Heterozygous genotype; Hom: Homozygous genotype; MAF: Minor Allele Frequency; H-W: *p*-value for the Hardy-Weinberg equilibrium analysis.

**Table 4:** (**A**). Alleles distribution and association study with response (BCVA improvement/worsening) at 1-month follow-up. (**B**). Alleles distribution and association study with response (BCVA improvement/worsening) at 6-months follow-up.

(**A**)									
**SNP** **Major > Minor**	**Allele**	**IMPROVEMENT**				**WORSENING**			
		**YES** **n (%)**	**NO** **n (%)**	**OR (95%CI)**	***p*** **-Value**	**YES** **n (%)**	**NO** **n (%)**	**OR ** **(95%CI)**	***p*** **-Value**
**1-Month Follow-Up**									
*CXCL8 * rs4073 A > T	A	70 (46.7)	34 (45.9)	1.03 (0.57–1.87)	0.919	9 (56.2)	95 (45.7)	1.53 (0.49–5.02)	0.414
	T	80 (53.3)	40 (54.1)			7 (43.8)	113 (54.3)		
*NRP1* rs2070296 C > T	T	22 (14.7)	12 (16.2)	0.89 (0.39–2.11)	0.761	1 (6.2)	33 (15.9)	0.35 (0.01–2.46)	0.302
	C	128 (85.3)	62 (83.8)			15 (93.8)	175 (84.1)		
*F13A1 * rs5985 C > A	C	118 (78.7)	58 (78.4)	1.02 (0.48–2.09)	0.961	11 (68.8)	165 (79.3)	0.57 (0.17–2.23)	0.320
	A	32 (21.3)	16 (21.6)			5 (31.2)	43 (20.7)		
*ARMS2 * rs10490924 G > T	G	113 (75.3)	54 (73.0)	1.13 (0.57–2.22)	0.703	13 (81.2)	154 (74.0)	1.52 (0.4–8.61)	0.523
	T	37 (24.7)	20 (27.0)			3 (18.8)	54 (26.0)		
*CFH * rs1061170 C > T	C	52 (34.7)	18 (24.3)	1.65 (0.85–3.3)	0.116	1 (6.2)	69 (33.2)	0.13 (0–0.91)	**0.025**
	T	98 (65.3)	56 (75.7)			15 (93.8)	139 (66.8)		
*VEGFA * rs3025040 C > T	C	130 (86.7)	68 (91.9)	0.57 (0.18–1.57)	0.251	14 (87.5)	184 (88.5)	0.91 (0.19–8.77)	0.908
	T	20 (13.3)	6 (8.1)			2 (12.5)	24 (11.5)		
(**B**)									
**SNP** **Major > Minor**	**Allele**	**IMPROVEMENT**				**WORSENING**			
		**YES** **n (%)**	**NO** **n (%)**	**OR (95%CI)**	***p*** **-Value**	**YES** **n (%)**	**NO** **n (%)**	**OR ** **(95%CI)**	***p*** **-Value**
**6-Months Follow-up**									
*CXCL8 * rs4073 A>T	A	74 (46.8)	30 (45.5)	1.06 (0.57–1.96)	0.850	13 (46.4)	91 (46.4)	1 (0.41–2.39)	1
	T	84 (53.2)	36 (54.5)			15 (53.6)	105 (53.6)		
*NRP1* rs2070296 C>T	T	27 (17.1)	7 (10.6)	1.74 (0.69–4.99)	0.218	3 (10.7)	31 (15.8)	0.64 (0.12–2.3)	0.482
	C	131 (82.9)	59 (89.4)			25 (89.3)	165 (84.2)		
*F13A1 * rs5985 C>A	C	125 (79.1)	51 (77.3)	1.11 (0.52–2.32)	0.760	23 (82.1)	153 (78.1)	1.29 (0.44–4.61)	0.623
	A	33 (20.9)	15 (22.7)			5 (17.9)	43 (21.9)		
*ARMS2 * rs10490924 G>T	G	124 (78.5)	43 (65.2)	1.95 (0.98–3.83)	0.037	17 (60.7)	150 (76.5)	0.47 (0.19–1.21)	0.073
	T	34 (21.5)	23 (34.8)			11 (39.3)	46 (23.5)		
*CFH * rs1061170 C>T	C	55 (34.8)	15 (22.7)	1.82 (0.9–3.8)	0.075	7 (25)	63 (32.1)	0.7 (0.24–1.84)	0.446
	T	103 (65.2)	51 (77.3)			21 (75)	133 (67.9)		
*VEGFA * rs3025040 C>T	C	140 (88.6)	58 (87.9)	1.07 (0.38–2.77)	0.877	23 (82.1)	175 (89.3)	0.55 (0.18–2.06)	0.270
	T	18 (11.4)	8 (12.1)			5 (17.9)	21 (10.7)		

OR: Odds ratio; CI: Confidence interval; NA: Not applicable; Bold: Significant results.

**Table 5:** (**A**). *ARMS2* (rs10490924) and *CFH* (rs1061170) genotype association with BCVA improvement/worsening at 1 month. (**B**). *ARMS2* (rs10490924) and *CFH* (rs1061170) genotype association with BCVA improvement/worsening at 6 months.

**(A)**								
**SNP**	**Genotype**	**IMPROVEMENT**						
		**YES** **n (%)**	**NO** **n (%)**	**Genetic Model** **(Reference)**	**OR (95%CI)**	***p*-Value**	**AIC**	**BIC**
*ARMS2* rs10490924 G>T	G/G	45 (59.2)	18 (50)	Codominant (GG) a	1.77 (0.77–4.05)	0.160	143	151.2
	T/G	24 (31.6)	17 (47.2)	Codominant (GG) b	0.36 (0.04–3.11)			
	T/T	7 (9.2)	1 (2.8)	Dominant (GG)	1.45 (0.65–3.22)	0.360	143.8	149.3
				Recessive (TT)	3.55 (0.42–30.01)	0.180	142.9	148.3
				Overdominant (TG)	0.52 (0.23–1.16)	0.110	142.1	147.6
				Log-additive	0.93 (0.50–1.75)	0.830	144.6	150
*CFH* rs1061170 C > T	T/T	32 (42.1)	21 (58.3)	Codominant (TT) c	0.57 (0.24–1.31)	0.230	143.7	151.8
	T/C	35 (46)	13 (36.1)	Codominant (TT) d	0.34 (0.07–1.72)			
	C/C	9 (11.8)	2 (5.6)	Dominant (TT)	0.52 (0.23–1.16)	0.110	142.1	147.5
				Recessive (CC)	2.28 (0.47–11.16)	0.270	143.5	148.9
				Overdominant (TC)	1.51 (0.67–3.42)	0.320	143.7	149.1
				Log-additive	1.74 (0.91–3.33)	0.084	141.7	147.1
**SNP**	**Genotype**	**WORSENING**						
		**YES** **n (%)**	**NO** **n (%)**	**Genetic Model**	**OR (95%CI)**	***p*-Value**	**AIC**	**BIC**
*ARMS2* rs10490924 G > T	G/G	5 (62.5)	58 (55.8)	Codominant (GG) a	1.09 (0.25–4.84)	0.540	62.4	70.5
	T/G	3 (37.5)	38 (36.5)	Codominant (GG) b	NA (0.00–NA)			
	T/T	0 (0)	8 (7.7)	Dominant (GG)	1.32 (0.30–5.82)	0.710	61.5	66.9
				Recessive (TT)	0.00 (0.00–NA)	0.270	60.4	65.8
				Overdominant (TG)	1.04 (0.24–4.61)	0.960	61.6	67.1
				Log-additive	0.67 (0.19–2.39)	0.520	61.2	66.7
*CFH* rs1061170 C > T	T/T	7 (87.5)	46 (44.2)	Codominant (TT) c	7.15 (0.85–60.45)	0.038	57.1	65.3
	T/C	1 (12.5)	47 (45.2)	Codominant (TT) d	NA (0.00–NA)			
	C/C	0 (0)	11 (10.6)	Dominant (TT)	8.83 (1.05–74.30)	**0.013**	**55.5**	**60.9**
				Recessive (CC)	0.00 (0.00–NA)	0.190	59.9	65.4
				Overdominant (TC)	0.17 (0.02–1.46)	0.053	57.9	63.3
				Log-additive	0.13 (0.02–1.03)	0.011	55.2	60.6
**(B)**								
**SNP**	**Genotype**	**IMPROVEMENT**						
		**YES** **n (%)**	**NO** **n (%)**	**Genetic Model** **(Reference)**	**OR (95%CI)**	***p*-Value**	**AIC**	**BIC**
*ARMS2* rs10490924 G > T	G/G	50 (62.5)	13 (40.6)	Codominant (GG) a	2.46 (1.03–5.91)	0.110	135.6	143.7
	G/T	25 (31.2)	16 (50)	Codominant (GG) b	2.31 (0.49–10.94)			
	T/T	5 (6.2)	3 (9.4)	Dominant (GG)	2.44 (1.05–5.63)	**0.035**	**133.6**	**139**
				Recessive (TT)	0.64 (0.14–2.87)	0.570	137.7	143.1
				Overdominant (GT)	0.45 (0.20–1.05)	0.065	134.6	140.1
				Log-additive	1.85 (0.97–3.51)	0.060	134.5	139.9
*CFH* rs1061170 C > T	T/T	34 (42.5)	19 (59.4)	Codominant (TT) c	0.66 (0.28–1.55)	0.012	131.2	139.4
	T/C	35 (43.8)	13 (40.6)	Codominant (TT) d	0.00 (0.00–NA)			
	C/C	11 (13.8)	0 (0)	Dominant (TT)	0.51 (0.22–1.16)	0.110	135.4	140.8
				Recessive (CC)	NA (0.00–NA)	**0.005**	**130.1**	**135.6**
				Overdominant (CT)	1.14 (0.49–2.61)	0.76	137.9	143.4
				Log-additive	0.45 (0.22–0.92)	0.021	132.7	138.2
**SNP**	**Genotype**	**WORSENING**						
		**YES** **n (%)**	**NO** **n (%)**	**Genetic Model**	**OR (95%CI)**	***p*** **-Value**	**AIC**	**BIC**
*ARMS2* rs10490924 G > T	G/G	4 (28.6)	59 (60.2)	Codominant (GG) a	0.24 (0.07–0.84)	0.067	85	93.1
	G/T	9 (64.3)	32 (32.6)	Codominant (GG) b	0.47 (0.05–4.86)			
	T/T	7 (7.1)	1 (7.1)	Dominant (GG)	0.26 (0.08–0.90)	0.025	83.4	88.8
				Recessive (TT)	1.00 (0.11–11.80)	1	88.4	93.8
				Overdominant (GT)	3.71 (1.15–11.98)	**0.024**	**83.3**	**88.8**
				Log-additive	0.48 (0.21–1.11)	0.089	85.5	90.6
*CFH* rs1061170 C > T	T/T	7 (50)	46 (46.9)	Codominant (TT) c	0.89 (0.29–2.76)	0.21	87.3	95.4
	T/C	7 (50)	41 (41.8)	Codominant (TT) d	NA (0.00–NA)			
	C/C	0 (0)	11 (11.2)	Dominant (TT)	1.13 (0.37–3.47)	0.83	88.4	93.8
				Recessive (CC)	0.00 (0.00–NA)	0.078	85.3	90.7
				Overdominant (CT)	1.39 (0.45–4.27)	0.570	88.1	93.5
				Log-additive	1.42 (0.57–3.52)	0.440	87.8	93.2

SNP: Single Nucleotide Polymorphism; OR: Odds Ratio; CI: Confidence Interval; AIC: Akaike information criterion; BIC: Bayesian information criterion; NA: Not applicable; Bold: Significant result. a: G/G vs. T/G; b: G/G vs. T/T; c: T/T vs. T/C; d: T/T vs. C/C.

## Data Availability

The data presented in this study are available on request from the corresponding author. The data are not publicly available due to containing clinical and personal information.

## Supplementary Materials

The following are available online at https://www.mdpi.com/article/10.3390/pharmaceutics13111973/s1, Table S1: Genotypes association with BCVA improvement/worsening at 1 month, Table S2: *CXCL8* (rs4073), *NRP1*(rs2070296), *F13A1* (rs5985) and *VEGFA* (rs3025040) genotype association with BCVA improvement/worsening at 6 months, Figure S1: Linkage disequilibrium analysis for VEGFA gene.
