# Supplementary material for: Genetic Polymorphisms Affecting Ranibizumab Response in High Myopia Patients
Source: Pharmaceutics. 2021 Nov 20;13(11):1973. doi: 10.3390/pharmaceutics13111973 (PMC8620862; doi:10.3390/pharmaceutics13111973)
Supplement: Supplementary file 1 [file pharmaceutics-13-01973-s001.zip › pharmaceutics-1393171-supplementary.pdf]

pharmaceutics MDPI

Supplementary Materials: Genetic Polymorphisms Affecting Ranibizumab Response in High Myopia Patients

David Blánquez-Martíne, Xando Díaz-Villamarín,*, Alba Antúnez-Rodríguez, Ana Pozo-Agundo, José Ignacio Muñoz-Ávila, Luis Javier Martínez-González and Cristina Lucía Dávila-Fajardo

Table S1: Genotypes association with BCVA improvement/worsening at 1 month.

SNP	Genotype	IMPROVEMENT						
		YES n (%)	NO n (%)	Genetic model (reference)	OR (95%CI)	p-value	AIC	BIC
CXCL8 rs4073	*T/T*	20 (26.3)	9 (25)	Codominant (TT) a	1.14 (0.44-2.93)	0.890	146.4	154.6
	T/A	41 (54)	21 (58.3)	Codominant (TT) b	0.89 (0.26-3.04)			
	A/A	15 (19.7)	6 (16.7)	Dominant (TT)	1.07 (0.43-2.66)	0.880	144.6	150.1
				Recessive (AA)	1.23 (0.43-3.49)	0.700	144.5	149.9
				Overdominant (TA)	0.84 (0.38-1.87)	0.660	144.5	149.9
				Log-additive	1.04 (0.57-1.89)	0.900	144.6	150.1
NRP1 rs2070296	*C/C*	55 (72.4)	25 (69.4)	Codominant (CC) c	1.27 (0.53-3.07)	0.400	144.8	153
	T/C	19 (25)	11 (30.6)	Codominant (CC) d	0.00 (0.00-NA)			
	T/T	2 (2.6)	0 (0)	Dominant (CC)	1.15 (0.48-2.75)	0.750	144.6.	150
				Recessive (TT)	NA (0.00-NA)	0.210	143.1	148.5
				Overdominant (TC)	0.76 (0.31-1.82)	0.540	144.3	149.7
				Log-additive	0.99 (0.45-2.19)	0.980	144.7	150.1
F13A1 rs5985	*C/C*	46 (60.5)	22 (61.1)	Codominant (CC) e	1.01 (0.44-2.32)	0.950	146.6	154.7
	A/C	27 (35.5)	13 (36.1)	Codominant (CC) f	0.70 (0.07-7.09)			
	A/A	3 (4)	1 (2.8)	Dominant (CC)	0.98 (0.43-2.20)	0.950	144.7	150.1
				Recessive (AA)	1.44 (0.14-14.33)	0.750	144.6	150
				Overdominant (AC)	0.97 (0.43-2.23)	0.950	144.7	150.1
				Log-additive	1.06 (0.52-2.15)	0.880	144.6	150.1

VEGFA rs3025040	*C/C*	57 (75)	30 (83.3)	Codominant (CC) g	0.63 (0.23-1.76)	0.450	145.1	153.2
	T/C	13 (23.7)	6 (16.7)	Codominant (CC) h	0.00 (0.00-NA)			
	T/T	1 (1.3)	0 (0)	Dominant (CC)	0.60 (0.22-1.66)	0.310	143.6	149.1
				Recessive (TT)	NA (0.00-NA)	0.380	143.9	149.3
				Overdominant (TC)	1.55 (0.56-4.32)	0.390	143.9	149.4
				Log-additive	1.71 (0.64-4.55)	0.270	143.4	148.9
		WORSENING						
		YES n (%)	NO n (%)	Genetic model	OR (95%CI)	p-value	AIC	BIC
CXCL8 rs4073	*T/T*	2 (25)	27 (26)	Codominant (TT) a	1.46 (0.23-9.23)	0.400	61.8	70
	T/A	3 (37.5)	59 (56.7)	Codominant (TT) b	0.44 (0.07-2.93)			
	A/A	3 (37.5)	18 (17.3)	Dominant (TT)	0.95 (0.18-5.00)	0.950	61.6	67.1
				Recessive (AA)	2.87 (0.63-13.09)	0.190	60	65.4
				Overdominant (TA)	0.46 (0.10-2.02)	0.290	60.5	66
				Log-additive	1.62 (0.54-4.83)	0.390	60.9	66.3
NRP1 rs2070296	*C/C*	7 (87.5)	73 (70.2)	Codominant (CC) c	2.78 (0.33-23.61)	0.500	62.2	70.4
	T/C	1 (12.5)	29 (27.9)	Codominant (CC) d	NA (0.00-NA)			
	T/T	0 (0)	2 (1.9)	Dominant (CC)	2.97 (0.35-25.19)	0.260	60.4	65.8
				Recessive (TT)	0.00 (0.00-NA)	0.580	61.3	66.8
				Overdominant (TC)	0.37 (0.04-3.14)	0.310	60.6	66
				Log-additive	0.34 (0.04-2.74)	0.250	60.3	65.7
F13A1 rs5985	*C/C*	3 (37.5)	65 (62.5)	Codominant (CC) e	0.32 (0.07-1.43)	0.230	60.7	68.9
	A/C	5 (62.5)	35 (33.6)	Codominant (CC) f	NA (0.00-NA)			
	A/A	0 (0)	4 (3.8)	Dominant (CC)	0.36 (0.08-1.59)	0.170	59.7	65.2
				Recessive (AA)	0.00 (0.00-NA)	0.440	61	66.5
				Overdominant (AC)	3.29 (0.74-14.55)	0.110	59.1	64.5
				Log-additive	1.82 (0.57-5.86)	0.320	60.7	66.1
VEGFA rs3025040	*C/C*	6 (75)	81 (77.9)	Codominant (CC) g	0.81 (0.15-4.32)	0.900	63.4	71.6

	T/C	2 (25)	22 (21.1)	Codominant (CC) h	NA (0.00-NA)			
	T/T	0 (0)	1 (1)	Dominant (CC)	0.85 (0.16-4.51)	0.850	61.6	67
				Recessive (TT)	0.00 (0.00-NA)	0.700	61.5	66.9
				Overdominant (TC)	1.24 (0.23-6.59)	0.800	61.6	67
				Log-additive	1.10 (0.23-5.34)	0.810	61.6	67.1

SNP: Single Nucleotide Polymorphism; OR: Odds Ratio; CI: Confidence Interval; AIC: Akaike information criterion; BIC: Bayesian information criterion; NA: Not applicable
a: T/T vs T/A; b: T/T vs A/A; c: C/C vs T/C; d: C/C vs T/T; e: C/C vs A/C; f: C/C vs A/A; g: C/C vs T/C; h: C/C vs T/T; i: G/G vs T/G; j: G/G vs T/T; k: T/T vs T/C; l: T/T vs C/C

Table S2: CXCL8 (rs4073), NRP1(rs2070296), F13A1 (rs5985) and VEGFA (rs3025040) genotype association with BCVA improvement/worsening at 6 months.

SNP	Genotype	IMPROVEMENT						
		YES n (%)	NO n (%)	Genetic model (reference)	OR (95%CI)	p-value	AIC	BIC
CXCL8 rs4073	*T/T*	20 (25)	9 (28.1)	Codominant (TT) a	0.84 (0.32-2.20)	0.940	139.9	148
	T/A	45 (56.2)	17 (53.1)	Codominant (TT) b	0.89 (0.26-3.04)			
	A/A	15 (18.8)	6 (18.8)	Dominant (TT)	0.85 (0.34-2.14)	0.730	137.9	143.3
				Recessive (AA)	1 (0.35-2.86)	1	138	143.4
				Overdominant (TA)	1.13 (0.50-2.58)	0.760	137.9	143.4
				Log-additive	1.07 (0.58-1.99)	0.82	138	143.4
NRP1 rs2070296	*C/C*	55 (68.8)	25 (78.1)	Codominant (CC) c	0.67 (0.25-1.76)	0.360	138	146.1
	T/C	23 (28.8)	7 (21.9)	Codominant (CC) d	0.00 (0.00-NA)			
	T/T	2 (2.5)	0 (0)	Dominant (CC)	0.62 (0.24-1.61)	0.310	137	142.4
				Recessive (TT)	NA (0.00-NA)	0.240	136.7	142.1
				Overdominant (TC)	1.44 (0.55-3.79)	0.450	137.4	142.9
				Log-additive	1.69 (0.68-4.19)	0.240	136.6	142.1
F13A1 rs5985	*C/C*	49 (61.2)	19 (59.4)	Codominant (CC) e	1.11 (0.47-2.61)	0.960	139.9	148.1
	A/C	28 (35)	12 (37.5)	Codominant (CC) f	0.86 (0.08-8.79)			
	A/A	3 (3.8)	1 (3.1)	Dominant (CC)	1.08 (0.47-2.50)	0.850	138	143.4
				Recessive (AA)	1.21 (0.12-12.06)	0.870	138	143.4
				Overdominant (AC)	0.90 (0.38-2.10)	0.800	138	143.4

				Log-additive	0.96 (0.47-1.99)	0.920	138	143.4
VEGFA rs3025040	*C/C*	63 (78.8)	24 (75)	Codominant (CC) g	1.31 (0.50-3.46)	0.610	139	147.2
	T/C	16 (20)	8 (25)	Codominant (CC) h	0.00 (0.00-NA)			
	T/T	1 (1.2)	0 (0)	Dominant (CC)	1.24 (0.47-3.24)	0.670	137.8	143.3
				Recessive (TT)	NA (0.00-NA)	0.410	137.3	142.8
				Overdominant (TC)	0.75 (0.28-1.98)	0.560	137.7	143.1
				Log-additive	0.88 (0.36-2.19)	0.790	137.9	143.4
		WORSENING						
		YES n (%)	NO n (%)	Genetic model	OR (95%CI)	p-value	AIC	BIC
CXCL8 rs4073	*T/T*	3 (21.4)	26 (26.5)	Codominant (TT) a	0.68 (0.17-2.72)	0.770	89.9	98
	T/A	9 (64.3)	53 (54.1)	Codominant (TT) b	1.10 (0.17-7.21)			
	A/A	2 (14.3)	19 (19.4)	Dominant (TT)	0.76 (0.20-2.92)	0.680	88.2	93.7
				Recessive (AA)	0.69 (0.14-3.36)	0.640	88.2	93.6
				Overdominant (TA)	1.53 (0.48-4.89)	0.470	87.9	93.3
				Log-additive	1.00 (0.43-2.32)	NA	88.4	93.8
NRP1 rs2070296	*C/C*	11 (78.6)	69 (70.4)	Codominant (CC) c	1.43 (0.37-5.55)	0.660	89.6	97.7
	T/C	3 (21.4)	27 (27.6)	Codominant (CC) d	NA (0.00-NA)			
	T/T	0 (0)	2 (2)	Dominant (CC)	1.54 (0.40-5.93)	0.520	88	93.4
				Recessive (TT)	0.00 (0.00-NA)	0.460	87.9	93.3
				Overdominant (TC)	0.72 (0.19-2.77)	0.620	88.2	93.6
				Log-additive	0.63 (0.17-2.26)	0.460	87.8	93.3
F13A1 rs5985	*C/C*	9 (64.3)	59 (60.2)	Codominant (CC) e	1.07 (0.33-3.44)	0.580	89.3	97.5
	A/C	5 (35.7)	35 (35.7)	Codominant (CC) f	NA (0.00-NA)			
	A/A	0 (0)	4 (4.1)	Dominant (CC)	1.19 (0.37-3.82)	0.770	88.3	93.7
				Recessive (AA)	0.00 (0.00-NA)	0.300	87.3	92.7
				Overdominant (AC)	1.00 (0.31-3.22)	NA	88.4	93.8
				Log-additive	0.76 (0.27-2.19)	0.610	88.1	93.6
VEGFA rs3025040	*C/C*	9 (64.3)	78 (79.6)	Codominant (CC) g	0.44 (0.13-1.46)	0.380	88.4	96.6

	T/C	5 (35.7)	19 (19.4)	Codominant (CC) h	NA (0.00-NA)			
	T/T	0 (0)	1 (1)	Dominant (CC)	0.46 (0.14-1.53)	0.220	86.9	92.3
				Recessive (TT)	0.00 (0.00-NA)	0.600	88.1	93.6
				Overdominant (TC)	2.31 (0.69-7.69)	0.190	86.7	92.1
				Log-additive	1.88 (0.62-5.72)	0.280	87.2	92.7

SNP: Single Nucleotide Polymorphism; OR: Odds Ratio; CI: Confidence Interval; AIC: Akaike information criterion; BIC: Bayesian information criterion; NA: Not applicable
a: G/G vs T/G; b: G/G vs T/T; c: T/T vs T/C; d: T/T vs C/C

Table S3: VEGFA haplotype association with response.

VEGFA haplotype association with response (n=112, crude analysis)								
IMPROVEMENT AT 1 MONTH								
rs25648	rs699947	rs3025000	rs1570360	rs3025040	rs2010963	Freq	OR (95% CI)	p-value
C	C	T	G	C	C	0,289	1,00	-
C	A	C	A	C	G	0,2732	2,16 (0,99-4,72)	0,057
C	C	C	G	C	G	0,1658	2,35 (0,94-5,87)	0,07
T	A	C	G	C	G	0,1419	0,58 (0,24-1,39)	0,23
C	C	T	G	T	C	0,0547	5,02 (0,63-40,08)	0,13
C	A	C	A	T	G	0,0214		
WORSENING AT 1 MONTH								
rs25648	rs699947	rs3025000	rs1570360	rs3025040	rs2010963	Freq	OR (95% CI)	p-value
C	C	T	G	C	C	0,2864	1,00	-
C	A	C	A	C	G	0,2691	0,37 (0,07-1,93)	0,24
C	C	C	G	C	G	0,1727	0,73 (0,15-3,55)	0,69
T	A	C	G	C	G	0,1394	1,11 (0,29-4,27)	0,88
C	C	T	G	T	C	0,0553		
IMPROVEMENT AT 6 MONTH								
rs25648	rs699947	rs3025000	rs1570360	rs3025040	rs2010963	Freq	OR (95% CI)	p-value
C	C	T	G	C	C	0,2897	1,00	-
C	A	C	A	C	G	0,272	0,99 (0,47-2,09)	0,99
C	C	C	G	C	G	0,167	2,64 (0,90-7,78)	0,081
T	A	C	G	C	G	0,1407	0,85 (0,36-2,02)	0,71
C	C	T	G	T	C	0,054	0,50 (0,11-2,22)	0,36
C	A	C	A	T	G	0,0227		
WORSENING AT 6 MONTH								
rs25648	rs699947	rs3025000	rs1570360	rs3025040	rs2010963	Freq	OR (95% CI)	p-value
C	C	T	G	C	C	0,2904	1,00	-
C	A	C	A	C	G	0,2726	0,41 (0,13-1,29)	0,24
C	C	C	G	C	G	0,165	0,15 (0,02-1,17)	0,69
T	A	C	G	C	G	0,1417	0,61 (0,19-2,00)	0,88

C	C	T	G	T	C	0,0533	3,08 (0,65-14,57)		0,16
C	A	C	A	T	G	0,022			

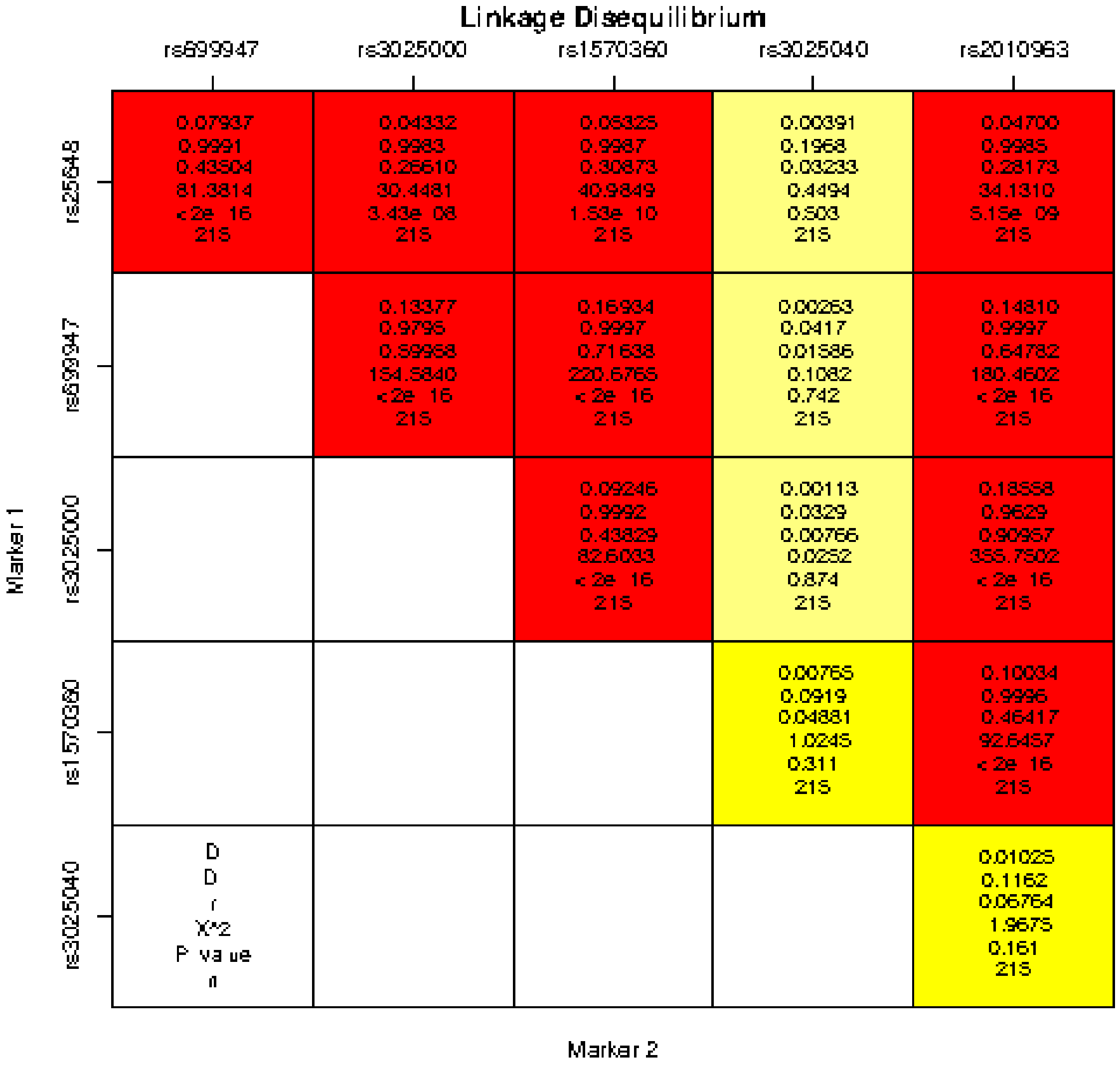

Figure S1: Linkage disequilibrium analysis for VEGFA gene.
